# Treatment optimization of beta-blockers in chronic heart failure therapy

**DOI:** 10.1038/s41598-020-72836-4

**Published:** 2020-09-28

**Authors:** Yirga Legesse Niriayo, Solomon Weldegebreal Asgedom, Gebre Teklemariam Demoz, Kidu Gidey

**Affiliations:** 1 Department of Clinical Pharmacy, School of Pharmacy, College of Health Sciences, Mekelle University, Mekelle, Tigray Ethiopia; 2 Clinical Pharmacy and Pharmacy Practice Unit, Departments of Pharmacy, College of Health Sciences, Aksum University, Aksum, Tigray Ethiopia

**Keywords:** Clinical pharmacology, Interventional cardiology

## Abstract

Although evidence based guidelines recommend optimal use of beta blockers in all patients with chronic heart failure unless contraindicated, they are often underutilized and/or prescribed below the recommended dosage in the majority of patients with heart failure. To our knowledge, however, the optimal use of beta-blockers in chronic heart failure is not investigated in Ethiopia. Therefore, the aim of our study was to investigate the utilization and optimization of beta blockers in the management of patients with chronic heart failure in Ethiopia. A prospective observational study was conducted among ambulatory patients with chronic heart failure in Ethiopia. We included adult patients with a diagnosis of heart failure with a baseline left ventricular ejection fraction < 40% who had been on follow-up for at least 6 months. Patients were recruited into the study during their appointment for medication refilling using simple random sampling technique. All patients were followed for at least 6 months to determine the optimal use of beta blockers. The optimal use of beta blockers was determined according to evidence based guidelines. After explaining the purpose of the study, we obtained written informed consent from all participants. Data were collected through patient interview and review of patients’ medical records. Binary logistic regression analysis was performed to identify factors associated with utilization of beta blockers. A total of 288 patients were included in the study**.** Out of the total, 67% of the patients were receiving beta blockers. Among the patients who received beta blockers, 34.2% were taking guideline recommended beta blockers while 65.8% were taking atenolol, which is not guideline recommended beta blocker. Among the patients who received guideline recommended beta blockers, only 3% were taking optimal dose. Prior hospitalization [Adjusted Odds ratio (AOR) 0.38, 95% confidence interval (CI) 0.19–0.76], dose of furosemide > 40 mg (AOR 0.39, 95% CI 0.20–0.76), ischemic heart disease (AOR 3.27, 95% CI 1.66–6.45), atrial fibrillation (AOR 4.41, 95% CI 1.38–14.13) were significantly associated with the utilization of beta-blockers. Despite proven benefit**,** beta blockers were not optimally used in most of the participants in this study. The presence of ischemic heart disease and atrial fibrillation were positively associated with the utilization of beta blockers while hospitalization and higher diuretic dose were negatively associated with the utilization of beta blockers. Clinicians should attempt to use evidence based beta blockers at guideline recommended target doses that have been shown to have morbidity and mortality benefit in chronic heart failure. Moreover, more effort needs to be done to minimize the potentially modifiable risk factors for underutilization of beta blocker in chronic heart failure therapy.

## Introduction

Heart failure (HF) is a significant contributor to cardiovascular disease burden that affects around 26 million people in the globe^1,2^. HF is a growing disease burden worldwide owing to increasing elderly population and comorbidity^1^. The burden of HF has been increasing in sub-Saharan Africa including Ethiopia^3,4^. Over the past decades, significant advances have been made in the therapies and prevention of HF^5^. However, HF remained the leading cause of morbidity and mortality^6^.

Beta-adrenergic receptors are the predominant G protein- coupled receptors subtypes that regulate the cardiac function and physiology^7,8^. Beta adrenergic receptors are activated by epinephrine and norepinephrine and the activation of adrenergic receptors has detrimental effects in patients with chronic HF^8,9^. Beta adrenergic receptors play an important role in the pathophysiology of heart disease and are common therapeutic targets sites for beta-blockers^9^. Beta blockers are drugs that bind to beta-adrenergic receptors and thereby block the binding of norepinephrine and epinephrine to these receptors^7,10^. The blockage of beta adrenergic receptors plays an important role in delaying the progression of HF. Therefore, the main benefit of beta-blockers in chronic HF is to block deleterious G protein over-activation in the heart^7,9,10^.

Beta blockers are the core component of standard therapy in chronic HF with reduced ejection fraction^11,12^. In patients with systolic HF, evidence-based beta-blockers (carvedilol, metoprolol, and bisoprolol) have been proved to have morbidity and mortality benefit in several randomized clinical trials^13–16^. The optimal use of beta-blockers has been demonstrated to improve symptoms, reduce hospitalizations, improve left ventricular function, and enhance survival in chronic HF patients with reduced ejection fraction^17,18^. Despite the proven benefit of beta blockers in chronic HF, they are often underutilized in actual clinical practice^19,20^.

In compensated HF patients with reduced ejection fraction, beta-blockers should be initiated at low dose and up-titrated slowly (usually doubling the dose every 2–4 weeks ) to a target dose or maximum tolerable dose^21^. Several studies reported that clinical benefits of beta-blockers in systolic HF are dose-dependent and a better benefit occurred at higher doses^22–25^. In the majority of patients with chronic HF, target doses of beta-blockers are achievable and tolerable^21,25,26^. Hence, beta–blockers should be titrated to attain a target dose or a maximum tolerable dose to get a maximum clinical benefit^11,12,27^.

According to evidence-based guidelines^11,12^, only carvedilol, metoprolol, and bisoprolol are recommended beta blockers in chronic HF with reduced ejection fraction. The recommended daily target doses of evidence based beta-blockers are 200 mg metoprolol, 10 mg bisoprolol, and 50 mg carvedilol^28^. Despite high rates of beta-blockers’ target doses are achieved in randomized clinical trials, the rates of target dose achievement in clinical practice remained low^26,29^. Suboptimal use of beta-blockers has been associated with poor treatment outcome in HF patients^26,30^.

Bet-blockers are well tolerated in the majority of patients with chronic HF^31,32^. More importantly, the target doses of beta-blockers are tolerable in the majority of HF patients with careful titration^28,33^. Hence, beta-blockers should be used at optimal dose in all patients with chronic HF unless contraindicated^11,12^. Conversely, they are often underutilized and/or prescribed below the recommended dosage in the majority of patients with HF^24,32,34^. To our knowledge, however, the optimal use of beta-blockers therapy in chronic HF is not investigated in Ethiopia. Therefore, our study investigated the utilization and optimization of beta blockers therapy in the management of patients with chronic HF in Ethiopia.

## Material and methods

### Study design and study setting

We conducted a prospective observational study at ambulatory care clinic of Jimma University Medical Center. Jimma University medical center is a teaching and referral hospital that provides service for both outpatient and inpatient. It is a major public hospital in southwest Ethiopia that serves for about 15 million people in the catchment area.

### Study participants

We included adult patients aged > 18 years with a diagnosis of HF with a baseline left ventricular ejection fraction < 40% who had been on follow up for at least 6 months. Patients with precautions and contraindications to the use of beta-blockers including hypotension (< 90/60mmg), bradycardia (< 60 beats per minute), asthma, decompensated heart failure and those patients with incomplete medical record were excluded from the study.

A sample of 355 was calculated using a single population proportion formula assuming 50% rate of optimal use of beta-blockers among patients with HF, 95% confidence level, 5% margin of error, and 10% of contingency for nonresponse rate. From a total of 355 participants approached, 67 patients were excluded from the study due to precaution/contraindication to beta-blockers [55] and incomplete medical record [12].

### Data collection procedure

We recruited patients into the study during their appointment for medication refilling using simple random sampling technique. We obtained written informed consent from all participants after we had explained the purpose of the study Data regarding socio-demographics were retrieved using face to face interview. Respective patients clinical and treatment related characteristics were retrieved from patients’ medical record using data abstraction checklist. In order to determine whether the doses of beta blockers are up-titrated to a target dose/ maximum tolerable dose or not, all patients were followed for at least 6 months.

The optimal use of beta blockers was determined according to evidence based guidelines^11,28^. Patients were said to be optimally treated if they received optimal dose of guideline-recommended beta blockerss (metoprolol/carvedilol). Beta blockers were said to be underutilized if they were not used by patients in the absence of contraindication. Patients were considered as receiving guideline-recommended beta-blocker therapy if they received a prescription of carvedilol, or metoprolol while they were considered as receiving non-recommended beta blocker if they received a prescription of atenolol. The dose of beta blockers was said to be optimal if it was given at guideline-recommended target dose or a maximum tolerable dose is given for the patients. Whereas, the dose was said to be suboptimal if the patient was taking any dose of beta blockers below the target dose in the absence of contraindications for up-titration. For metoprolol, the optimal dose was considered if the dose was = 200 mg daily or maximum tolerable dose and for carvedilol, the dose was said to be optimal if it was ≥ 50 mg daily or maximum tolerable dose.

### Data analysis

We used EPI data management (version 4.2.0) and the Statistical Package for the Social Science (SPSS version 21.0) to record and analyze the data, respectively. We computed the frequency; mean (standard deviation), and median (interquartile) of categorical and continuous variables using descriptive analysis. We checked multicollinearity among predictor variables using variance inflation factor (VIF) and none was collinear. Univariable logistic regression analysis was performed to determine the association of each independent variable with utilization of beta blockers. Furthermore, multivariable binary logistic regression model was done to identify predictors of beta blockers utilization. A p value of < 0.05 was considered statistically significant in all analyses.

### Ethics approval and informed consent

Approval for this study was obtained from the institutional review board of Jimma University, College of Health and Medical Sciences. We fully explained the purpose and protocol of the study to all participants included in the study and written informed consent was obtained from each participant. The personal information was entirely confidential and protected. All methods were performed in accordance with the approved institutional guidelines.

## Results

### Sociodemographic and clinical characteristics

A total of 288 patients were included in the study. The mean [± standard deviation (SD)] age of the patients was 52.7 ± 14.7 years and 55.9% were males. Two-thirds of the participants were rural dwellers and more than half (57.3%) were unable to write and read (Table 1).

**Table 1:** Sociodemographic related characteristics of HF patients (n = 288).

Variables	n (%)
Gender, male	161 (55.9)
**Age in years**	
< 65	215 (74.7)
≥ 65	73 (25.3)
Residence, rural	189 (65.6)
**Educational level**	
Unable to write and read	165 (57.3)
Primary education	47 (16.3)
Secondary education	30 (10.4)
College and above	46 (16)
**Marital status**	
Married	222 (77.1)
Single	24 (8.3)
Divorced	18 (6.3)
Widowed	24 (8.3)
**Monthly income in Ethiopian birr**	
≤ 1500	150 (52.1)
> 1500	138 (47.9)

#### Clinical related characteristics

In this study, the majority of patients were in New York Heart Association (NYHA) class III (52.1%) and II (43.4%). More than half (58%) of the participants had been hospitalized one or more times during the last year. Two-thirds (67%) of the participants had two or more comorbidities. Ischemic heart disease (52.8%), hypertension (29.5%), and valvular heart disease (16.3%) were commonly identified comorbid diseases. The mean arterial blood pressure (MAP) was 87.6 (± 10.2) mmHg and the mean ejection fraction (EF) was 27.5 (± 6.5) % (Table 2).

**Table 2:** Clinical related characteristics of HF patients (n = 288).

Characteristics	n (%)
**NYHA class**	
I	13 (4.5)
II	125 (43.4)
III	150 (52.1)
**Hospitalization**	
No	121 (42)
Yes	167 (58)
Mean MAP (SD)	87.6 (10.2)
Mean EF (SD)	27.5% (6.5)
**Number of comorbidities**	
< 2	95 (33)
≥ 2	193 (67)
**Common comorbidities**	
Ischemic heart disease	152 (52.8)
Hypertension	85 (29.5)
Valvular heart diseases	47 (16.3)
Atrial fibrillation	43 (14.9)
Diabetes mellitus	25 (8.7)
Hyperthyroidism	23 (8)
Chronic kidney disease	12 (4.2)

### Treatment related characteristics

The mean (SD) duration of treatment was 2.8 (2.1) years. The mean (± SD) number of medications per patient was 4.3 ± 1.1 and 43.1% of the participants were taking five more medications. Angiotensin converting enzyme inhibitors (82.6%), loop diuretics (77.8%), and beta-blockers (67%) were most frequently used HF medications. More than half (58%) of the patients were receiving a combination of angiotensin converting enzyme inhibitors and beta blockers (Table 3).

**Table 3:** Treatment related characteristics of HF patients (n = 288).

Characteristics	n (%)
**Duration of treatment**	
≤ 2 year	159 (55.2)
> 2 year	129 (44.8)
**Number of medications**	157 (51)
< 5	164 (56.9)
≥ 5	124 (43.1)
**Frequently used medications**	
Angiotensin converting enzyme inhibitors	238 (82.6)
Furosemide	224 (77.8)
Beta-blockers	193 (67)
Angiotensin converting enzyme inhibitors + Beta-blockers	167 (58)
Antiplatelets	200 (69.4)
Statins	145 (50.3)
Spironolactone	50 (17.4)
Digoxin	48 (16.7)

### Utilization and dosing of beta-blockers in heart failure patients

Out of the total, 193(67%) participants received beta-blockers. Among the patients who received beta-blockers, only 66(34.2%) were taking evidence based beta-blockers while 127(65.8%) were taking atenolol, which is not evidence based beta blocker. Among the patients who took evidence based beta-blockers (66), only 2(3%) were receiving optimal dose. Of the total patients who received evidence based beta blockers, 7(10.6%) reached greater than or equals to 50% of the guideline recommended target dose. The mean daily doses of metoprolol, carvedilol, and atenolol that were taken by the participants were 29.8 mg, 19.1 mg, and 30.3 mg, respectively (Table 4).

**Table 4:** Type and dose of beta-blockers used in heart failure patients (n = 193).

Variables	Medications		
	Metoprolol	Carvedilol	Atenolol*
Number of patients on the medication (%)	50 (25.9)	16 (8.3)	127 (65.8)
Number of patients on optimal/target dose (%)	0	2 (3)	–
Number of patients on 50- < 100% target dose (%)	2 (3)	3 (4.5)	–
Number of patients on < 50%) target dose (%)	48 (72.7)	11 (16.7)	–
Mean (SD) daily dose (mg/d)	29.8 (16.1)	19.1 (13.2)	30.3 (14.3)
Median (IQR) dose received (mg)	25 (25)	12.5 (12.5–25)	25 (25)
Minimum dose used (mg/d)	12.5	6.25	12.5
Maximum dose used (mg/d)	100	50	100

*SD* standard deviation, *IQR* interquartile.

*The optimal/target dose of atenolol in heart failure is unknown as it not approved for use by evidence based guidelines.

### Factors associated with the utilization of Beta-blockers

We performed a univariable logistic regression analysis to compare HF patients who were receiving beta-blockers and not receiving beta-blockers using the socio-demographic, clinical and treatment-related characteristic. Accordingly, hospitalization (Crude Odds ratio (COR) 0.42, 95% confidence interval (CI) 0.25–0.71), dose of furosemide > 40 mg (COR 0.35, 95% CI 0.20–0.63), ischemic heart disease (COR 2.31, 95% CI 1.40–3.82), atrial fibrillation (COR 5.91, 95% CI 1.78–19.63) and number of medication ≥ 5 (COR 1.68, 95% CI 1.01–2.79) were significantly associated with the use of beta-blockers. Furthermore, variables with P < 0.25 in the univariable analysis were re-entered into the multivariable logistic regression model. The full model containing all predictors was statistically significant (Chi-square = 48.610, degree of freedom (*df*) = 8, *P* < 0.001). According to multivariable logistic regression analysis, hospitalization (Adjusted Odds ratio (AOR) 0.38, 95% confidence interval (CI) 0.19–0.76), dose of furosemide > 40 mg (AOR 0.39, 95% CI 0.20–0.76), ischemic heart disease (AOR 3.27, 95% CI 1.66–6.45), atrial fibrillation (AOR 4.41, 95% CI 1.38–14.13) were significantly associated with the use of beta-blockers (Table 5).

**Table 5:** Factors associated with the utilization of beta-blockers (n = 288).

Variables	Beta-blockers use		COR (95% CI)	*p* value	AOR (95%CI)	*p* value
	No, n (%)	Yes, n (%)				
**Age category**						
< 65	66 (69.5)	149 (77.2)	1	1	1	1
≥ 65	29 (30.5)	44 (22.8)	0.67 (0.39–1.17)	0.158	0.60 (0.30–1.23)	0.165
**Duration of treatment**						
≤ 2 year	47 (49.5)	112 (58)	1	1	1	1
> 2 year	48 (50.5)	81 (42)	0.71 (0.43–1.16)	0.170	0.78 (0.42–1.48)	0.449
**Hospitalization in the last one year**						
No	27 (28.4)	94 (48.7)	1	1	1	1
Yes	68 (71.6)	99 (51.3)	0.42 (0.25–0.71)	0.001	0.38 (0.19–0.76)	0.006
**Number of medications**						
< 5	62 (65.3)	102 (52.8)	1	1	1	1
≥ 5	33 (34.7)	91 (47.2)	1.68 (1.01–2.79)	0.046	1.80 (0.93–3.49)	0.080
**Ischemic heart disease**						
No	58 (61.1)	78 (40.4)	1	1	1	1
Yes	37 (38.9)	115 (59.6)	2.31 (1.40–3.82)	0.001	3.27 (1.66–6.45)	0.001
**Hypertension**						
No	62 (65.3)	141 (73.1)				
Yes	33 (34.7)	52 (26.9)	0.69 (0.41–1.18)	0.174	0.71 (0.36–1.40)	0.321
**Atrial fibrillation**						
No	91 (95.8)	154 (79.8)	1	1	1	1
Yes	4 (4.2)	39 (20.2)	5.91 (1.78–19.63)	0.004	4.41 (1.38–14.13)	0.013
**Dose of furosemide**						
≤ 40 mg	38 (48.1)	105 (72.4)	1	1	1	1
> 40 mg	41 (51.9)	40 (27.6)	0.35 (0.20–0.63)	< 0.001	0.39 (0.20- 0.76)	0.005

*COR* crude odds ratio, *AOR* adjusted odds ratio, *CI* confidence interval.

## Discussion

Although beta-blockers were traditionally thought to be contraindicated, they have consistently been shown to reduce morbidity and mortality in chronic HF with reduced ejection fraction^29,35^. Evaluation of utilization and optimization of beta blockers in chronic heart failure management is crucial to designing programs for future intervention. In the current study, we therefore investigated the utilization and optimization of beta-blockers among patients with HF.

In our study, 67% of the patients received beta blockers, which is in line with a study conducted in France, 65%^36^. Besides, 58% of the patients received a combination of beta blockers and angiotensin converting enzyme inhibitors, which is also consistent with France study, 61%^36^. Despite beta blockers are recommended to be used in all patients with HF with reduced ejection fraction unless contraindicated, one-third of the participants were not receiving beta blockers without any reason in the present study. In agreement with our study, beta blockers were underutilized in other similar studies^20,26,36,37^. Surprisingly, among the patients who received beta blockers, only one-third received guideline-recommended beta blockers. Based on the evidence based clinical practice guidelines^11,12,28^, metoprolol, carvediolol, and bisoprolol are the only recommended beta blockers in HF with reduced ejection fraction. In contrast, two-thirds of the participants were receiving another beta blocker (atenolol), which is not guideline-recommended beta blocker. The possible justification for the high prescription rate of atenolol might be due to the affordability issue as the evidence based beta blockers (metoprolol/carvedilol) are by far more expensive than atenolol. Furthermore, it could be attributed to the lack of availability of the evidence based beta blockers, absence of HF treatment guideline, and lack of awareness of medical practitioners about the differences between the evidence based beta blockers and other beta blockers in our setting.

In our study, the presence of ischemic heart disease and atrial fibrillation were positively associated with the utilization of beta blockers. This could be justified that atrial fibrillation and ischemic heart disease are additional indications of beta blockers^38–40^. Hence, this may initiate medical practitioners to prescribe beta-blockers. Beta blockers have been shown to reduce hospitalization in several studies^17,41–43^. Similarly, hospitalization was negatively associated with the use of beta blockers in the current study. Moreover, high dose of furosemide was negatively associated with the utilization of beta blockers. Consistent with our finding, the increased dose of furosemide has been reported to affect the use of beta blockers in chronic HF management^44^. Therefore, medical practitioners should prescribe diuretics with careful consideration taking into account their negative impact on the use of guideline-recommended drugs that have survival benefit. More importantly, the dose of furosemide needs to be optimized to allow utilization of beta blockers.

For the better outcome, beta blockers should be titrated up to a guideline-recommended target dose or maximum tolerable dose^12,21,34^. More importantly, the target doses are achievable in the majority of patients with chronic heart HF^31,33^. Despite guideline recommendations, most of the participants were receiving suboptimal dose in our study. In agreement to our study, most of the patients were receiving suboptimal dose in other similar studies^20,36,41^. In the present study, only 10.6% reached greater than or equals to 50% of the guideline-recommended target dose, which is quite different from the study conducted in France, 56%^26^. This could be attributed to the difference in medical practitioners’ expertise and the poor awareness of dose titration practice of beta blockers as observed in this study. Therefore, more effort needs to be done to up-titrate beta blockers to target dose or maximum tolerable dose.

## Strength and limitation of the study

The strength of our study is the prospective collection of data, which enables for accurate evaluation of the data related to treatment, clinical and laboratory parameters. Our study is the first to have full report on treatment optimization of beta blockers in Ethiopia. Hence, this finding may enhance future intervention to optimize beta-blockers use in patients with chronic HF.

Although we have attempted to assess various factors that might influence the utilization of beta blockers, we did not assess the impact of health professionals’ level of knowledge on the utilization of beta blockers. This study may not provide adequate evidence regarding the cause-effect relationship of beta-blocker utilization and its risk factors due to the inherent characteristics of cross-sectional study. Our study is a single center study; therefore, it might not be generalized to the general population. Moreover, as the findings of this study could be affected by the differences in participants’ characteristics, disease distribution, healthcare infrastructure, and methods employed, they should be extrapolated to other countries with caution.

## Conclusion

Despite the proven benefit in chronic HF, beta blockers were not optimally used in most of the participants in this study. The presence of ischemic heart disease and atrial fibrillation were positively associated with the utilization of beta blockers while hospitalization and higher diuretic dose were negatively associated with the utilization of beta blockers. Clinicians should attempt to use evidence based beta blockers at guideline recommended target doses that have been shown to have morbidity and mortality benefit in chronic heart failure. Furthermore, more efforts need to be done to minimize the potentially modifiable risk factors for underutilization of beta blocker in chronic heart failure therapy. More importantly, the dose of furosemide needs to be optimized to allow utilization of beta blockers.

## Data Availability

The dataset of this article is accessible on reasonable request from the corresponding author.
